# Long-Term Effectiveness and Safety of Femoropopliteal Drug-Coated Balloon Angioplasty : 5-Year Results of the Randomized Controlled EffPac Trial

**DOI:** 10.1007/s00270-022-03265-1

**Published:** 2022-09-11

**Authors:** Ulf Teichgräber, Thomas Lehmann, Maja Ingwersen, René Aschenbach, Thomas Zeller, Klaus Brechtel, Erwin Blessing, Michael Lichtenberg, Peter von Flotow, Britta Heilmeier, Sebastian Sixt, Steffen Brucks, Christian Erbel, Ulrich Beschorner, Michael Werk, Vicenç Riambau, Andreas Wienke, Christof Klumb, Markus Thieme, Dierk Scheinert

**Affiliations:** 1 Department of Diagnostic and Interventional Radiology, Friedrich-Schiller-University, Jena University Hospital, Am Klinikum 1, 07747 Jena, Germany; 2 Center for Clinical Studies, Friedrich-Schiller-University, Jena University Hospital, Jena, Germany; 3 University Heart Center Freiburg, Bad Krozingen, Bad Krozingen, Germany; 4 Department Radiology, MVZ GmbH Berlin-Tiergarten, Berlin, Germany; 5 SRH Clinic Karlsbad-Langensteinbach, Karlsbad, Germany; 6 Clinic of Angiology Arnsberg, Arnsberg, Germany; 7 Department of Angiology, Westpfalz-Clinic, Kusel, Germany; 8 Gefäßpraxis Im Tal, Munich, Germany; 9 Gefäßpraxis Biel/Bienne AG, Biel, Germany; 10 Angiologikum Hamburg, Hamburg, Germany; 11 Department of Cardiology, Angiology, Pneumology, Heidelberg University Hospital, Heidelberg, Germany; 12 Department of Radiology, Martin-Luther-Hospital, Berlin, Germany; 13 Division of Vascular Surgery, Cardiovascular Institute, Hospital Clínic, University of Barcelona, Barcelona, Spain; 14 Institute for Medical Epidemiology, Biostatistics, and Informatics, Martin-Luther-University Halle-Wittenberg, Halle, Germany; 15 Department of Angiology, Cardiology, Diabetology, Regiomed-Vascular Center, Sonneberg, Germany; 16 Department of Angiology, University Hospital Leipzig, Leipzig, Germany

**Keywords:** Angioplasty, Drug-coated balloon, Femoropopliteal, Paclitaxel

## Abstract

**Purpose:**

This study aimed to assess 5-year effectiveness and safety of femoropopliteal angioplasty with the Luminor® 35 drug-coated balloon (DCB).

**Materials and Methods:**

The EffPac trial was a prospective, multicenter, randomized controlled trial that enrolled 171 patients of Rutherford category 2 to 4 with medium length femoropopliteal lesions. Patients were allocated 1:1 to either Luminor® 35 DCB angioplasty or plain old balloon angioplasty (POBA). Assessment at 5 years included primary patency, freedom from clinically driven target lesion revascularization (CD-TLR), clinical improvement, and target limb amputation. Long-term vital status was ascertained in 97.1% of the participants.

**Results:**

*Kaplan*–Meier curves at 5 years demonstrate a primary patency of 61.4% after DCB angioplasty and 53.5% after POBA (log-rank *p* = 0.040) with a decreasing difference throughout the observation period. Freedom from TLR was 82.1% and 73.7%, respectively (log-rank *p* = 0.050). Incidence of primary clinical improvement was similar between groups (61% DCB vs. 64% POBA, *p* = 0.94). Major target limb amputation was necessary in one POBA-group participant. Freedom from all-cause death at 5 years was 88.5% after DCB and 86.0% after POBA (log-rank *p* = 0.34).

**Conclusions:**

Primary patency after femoropopliteal DCB angioplasty remained superior to POBA throughout 5 years, however, with decreasing difference. Clinical improvement, freedom from TLR, and all-cause mortality were similar between groups over the long term. (Effectiveness of Paclitaxel-Coated Luminor® Balloon Catheter Versus Uncoated Balloon Catheter in the Superficial Femoral Artery [EffPac]; NCT02540018).

**Supplementary Information:**

The online version contains supplementary material available at 10.1007/s00270-022-03265-1.

## Introduction

Drug-coated balloon (DCB) angioplasty using the antiproliferative effect of paclitaxel has been shown to reduce late lumen loss (LLL) and prevent restenosis more successfully than plain old balloon angioplasty (POBA) [1, 2]. However, effect size differs considerably across DCB types [3] and 5-year results are provided for only a few. In addition, a safety signal indicated increased long-term all-cause mortality with paclitaxel-eluting devices [4] and gave rise to a fierce debate that remains unsolved to this day [5].

Until now, data from randomized controlled trials throughout 5 years are available from the THUNDER [6], the IN.PACT SFA [7], the AcoArt [8], and the LEVANT studies [9]. The THUNDER, IN.PACT SFA, and AcoART studies found superior freedom from clinically driven target lesion revascularization (CD-TLR) after DCB compared to plain old balloon angioplasty (POBA). However, in THUNDER, neither Kaplan–Meier estimates nor information on precision are provided on primary patency. At 5 years, the LEVANT studies did only report on mortality but not on effectiveness. None of the above-mentioned studies found significant differences in all-cause mortality between DCB and POBA throughout 5 years.

The aim of the present EffPac long-term evaluation was to assess effectiveness and safety of femoropopliteal angioplasty with the Luminor® 35 (iVascular, Barcelona, Spain) DCB. In the EffPac trial, vital status at 5 years was obtained from almost all participants.

## Materials and Methods

### Study Design and Population

**Fig. 1:**
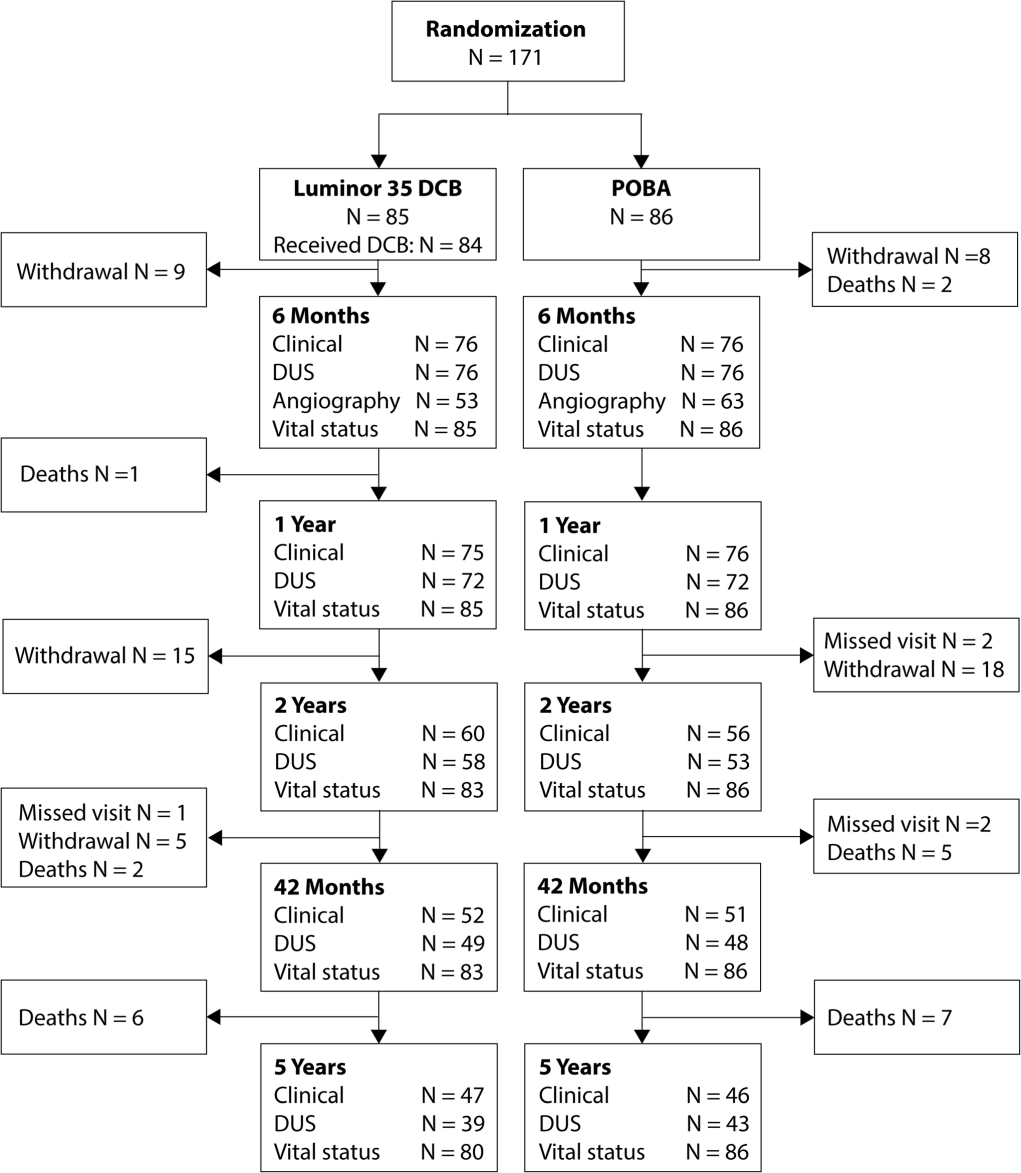
Participant flow in the EffPac trial through the 5-year follow-up. One hundred seventy-one patients were randomized 1:1 into groups that underwent Luminor 35 DCB angioplasty or plain old balloon angioplasty for the treatment of femoropopliteal lesions and were followed up through 60 months. Withdrawal was due to withdrawal of consent or to other reasons for loss to follow up *DCB* drug-coated balloon, *DUS* duplex ultrasonography, *POBA* plain old balloon angioplasty

The EffPac trial was a prospective, multicenter, participant- and core laboratory-blinded, randomized controlled study to compare Luminor® 35 DCB angioplasty with POBA in patients with femoropopliteal artery disease of Rutherford category 2–4. The detailed study design and eligibility criteria were published previously [1, 10] (Supplementary Table 1). Patients of Rutherford category 2–4 and single femoropopliteal target lesions of ≤ 15 cm were enrolled at 11 German sites between September 2015 and December 2016. Participants were allocated 1:1 to either DCB or POBA. Follow-ups included clinical and ultrasonographic examination at 6, 12, 24, 42, and 60 months. In addition, upon request of the Federal Institute for Drugs and Medical Devices (BfArM, Bonn, Germany) that had been addressed to all ongoing studies on paclitaxel-coated devices in July 2019, we attempted to obtain the vital status of all participants by telephone within 5 years after the intervention (Fig. 1) [11]. Adverse events were reviewed by an independent data safety monitoring board and angiographic and ultrasonographic findings were adjudicated by blinded, independent core laboratory staff members (CoreLab Black Forest, Bad Krozingen, Germany). The study complied with the Declaration of Helsinki and Good Clinical Practice Guidelines. The Protocol was approved by the responsible ethic committees of all investigational sites and all participants provided written informed consent. The EffPac trial is registered with ClinicalTrials.gov: NCT02540018.

### Study Intervention

Detailed description of the study intervention was provided earlier [1, 10, 12]. Briefly, patients were randomized only after the lesion had been crossed endoluminally with a guidewire and successfully pre-dilated with a standard balloon for 60 ± 10 s in both study arms. Study device was the CE-marked Luminor® 35 DCB catheter (iVascular S.L.U., Barcelona, Spain). This DCB is coated with paclitaxel at a surface concentration of 3 µg/mm^2^ and an organic ester excipient. Control devices were CE-marked uncoated balloon catheters. In case of dissection, prolonged balloon inflation was required. Bailout stenting was permitted at the discretion of the investigator. Participants received acetylsalicylic acid and clopidogrel for at least 4 weeks after the intervention.

### Study Outcome Measures

At 5 years, secondary long-term outcome measures included primary patency, freedom from CD-TLR, freedom from any target vessel revascularization (TVR), incidence of clinical and hemodynamic improvement, incidence of target limb amputation, and freedom from all-cause death. Primary patency was defined as absence of > 50% diameter restenosis of the target lesion by angiography or a peak systolic velocity ratio of < 2.4 by duplex ultrasonography without the need for TLR. Restenosis had to be adjudicated by the core laboratory. Any surgical or endovascular revascularization of the target lesion on grounds of symptoms was considered CD-TLR. Clinical improvement referred to improvement by at least one Rutherford category and hemodynamic improvement to an increase of the ankle-brachial index (ABI) by ≥ 0.15 or to 0.9. Primary improvement occurred if no TLR was needed, whereas secondary improvement also included participants who improved only after TLR. A post-hoc subgroup analysis on loss of primary patency on age, gender, diabetes/non-diabetes, smoker/non-smoker, lesion length, chronic total occlusion, calcification, dissection, and distal runoff vessels was conducted to assess interaction with the treatment effect.

### Statistical Analysis

Categorical data are presented as counts and percentages and were compared with Fisher’s exact- or Chi-square test when unpaired and with Wilcoxon signed-rank test when paired. Continuous data are described as means with standard deviations and compared with Student t or Mann–Whitney U test. Kaplan–Meier analysis was applied to provide time point estimates of primary patency, freedom from CD-TLR, freedom from TVR, and freedom from all-cause death, and to compare groups using the log-rank test. Sixty-five months were set as cutoff for the analyses. The level of statistical significance was set at *p* < 0.05. Post-hoc subgroup analysis on loss of primary patency was conducted with Cox proportional regression analysis that was adjusted for centers. Due to multiple testing in the subgroup analysis, the significance level was lowered to 0.006. Data were analyzed with SAS 9.4 (SAS Institute, Cary, NC, USA) and XLSTAT (Version 2015.6.01.24026, Addinsoft, Paris, France).

## Results

### Study Population

We randomized 171 subjects to either DCB angioplasty (*n* = 85, of those 84 underwent DCB angioplasty) or POBA (*n* = 86). At 5 years, 55% of the DCB group participants and 53% of the POBA group participants completed the clinical follow-up, and 46% and 50% the DUS follow-up, respectively. Vital status was ascertained in 94% of DCB group-, and in 100% of the POBA group participants (Fig. 1).

Participants were mainly patients with intermittent claudication with medium length femoropopliteal lesions (DCB group 5.9 cm, POBA group 5.6 cm). Twenty-three percent of the lesions were totally occluded and 49% severely or moderately calcified. Pre-dilation was conducted in 99% of the lesions in both treatment groups (Table 1). Detailed patient, lesion, and procedure characteristics were presented earlier [10]. The results of the post-hoc subgroup analysis on loss of primary patency are shown in Fig. 3.

**Table 1:** Participant, lesion, and procedure characteristics

	Luminor 35 DCB (*N* = 85)	POBA (*N* = 86)	*P* Value
Age, *y*	68 ± 8	68 ± 9	0.96
Male	51 (60)	60 (70)	0.20
Diabetes mellitus	31 (36)	35 (41)	0.64
Hypertension	74 (87)	73 (85)	0.83
Dyslipidemia	60 (71)	59 (69)	> 0.99
Current smoker	34 (40)	37 (43)	0.86
Rutherford category			0.53
2	13/85 (15)	18/85 (21)	
3	69/85 (81)	66/85 (78)	
4	2/85 (2)	1/85 (1)	
5	1/85 (1)	0	
Ankle-brachial index	0.73 ± 0.23	0.74 ± 0.23	0.78
Lesion length, mm	59 ± 43	56 ± 39	0.60
CTO	17 (20)	22 (26)	0.47
Lesion calcification^a^			0.23
Severe	3 (4)	10 (12)	
Moderate	36 (42)	35 (41)	
Pre-dilation	84 (99)	85 (99)	> 0.99
Bailout stenting	13 (15)	16 (19)	0.68

Data are mean ± standard deviation or counts (percentage)

^a^Severity of calcification upon visual assessment by the investigator

*DCB* drug-coated balloon, *POBA* plain old balloon angioplasty, *CTO* chronic total occlusion

### Effectiveness Outcomes Through 5 Years

**Fig. 2:**
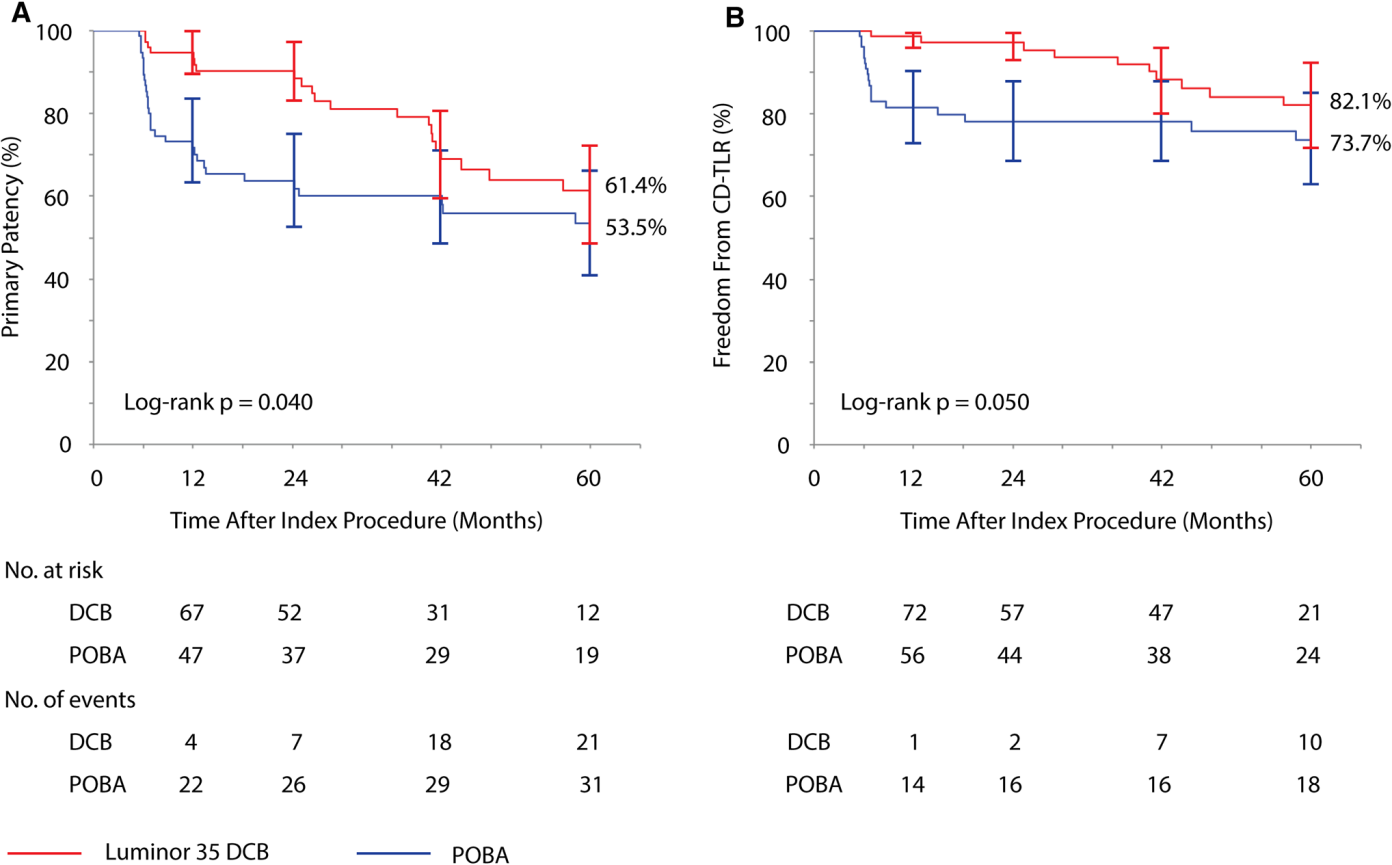
Treatment effect of Luminor 35 DCB angioplasty in femoropopliteal lesions through 5 years. **A** Primary patency at 5 years was achieved significantly more often with Luminor 35 DCB angioplasty than with standard balloon angioplasty and **B** freedom from clinically driven target lesion revascularization did not differ significantly between groups. At 60 months, standard error was 6.9% and 6.4% for primary patency and 5.2% and 5.5% for CD-TLR in the DCB and POBA group, respectively. Bars represent 95% confidence intervals. *DCB* drug-coated balloon, *POBA* plain old balloon angioplasty, *CD-TLR* clinically driven target lesion revascularization

**Fig. 3:**
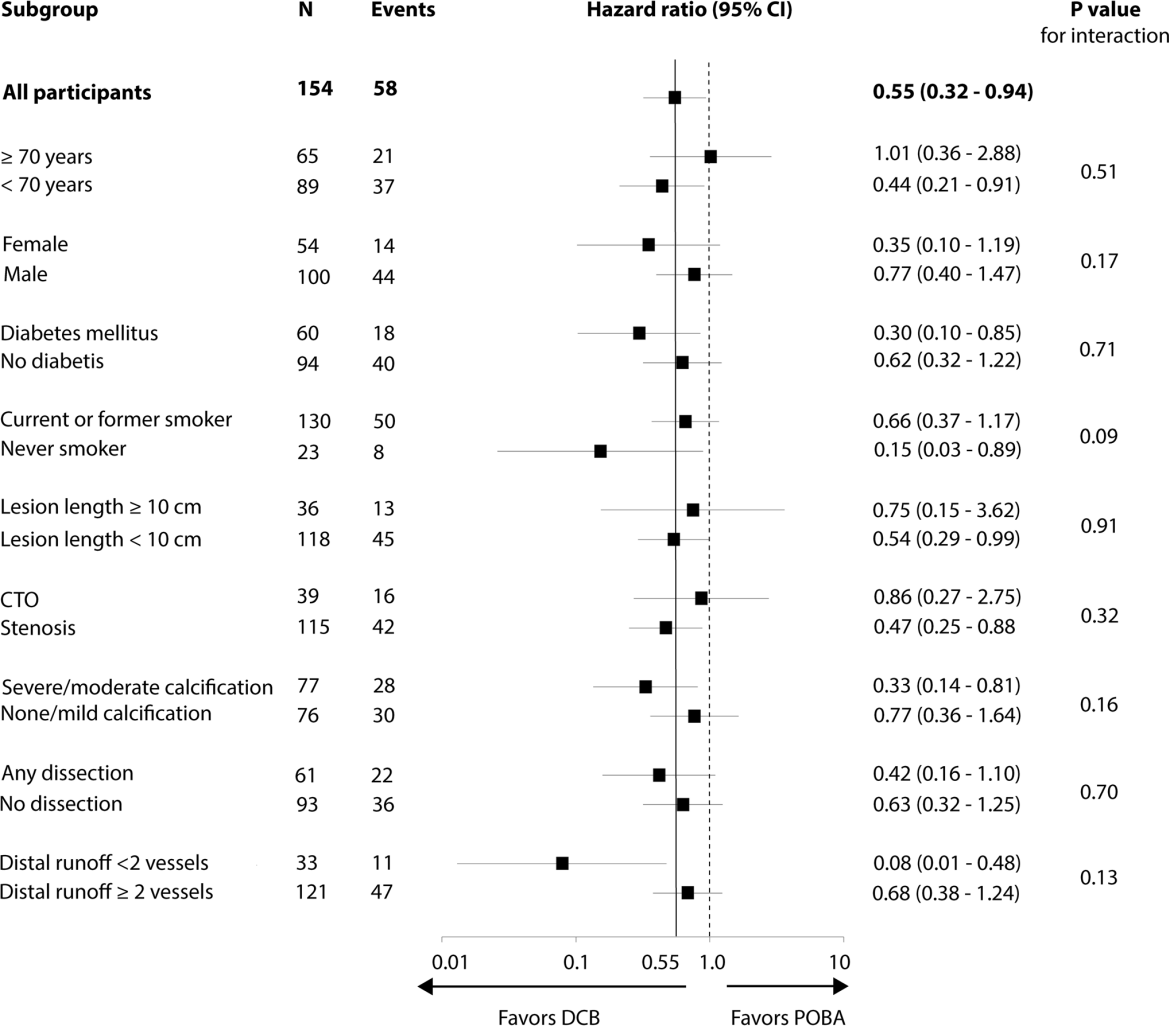
Post-hoc analysis of loss of primary patency by subgroups. Hazard ratios were determined over a period of 5 years and adjusted for study centers. The dotted line shows the no-effect point and the continuous line the overall treatment effect. *DCB* drug-coated balloon, *CTO* chronic total occlusion, *POBA* plain old balloon angioplasty

Superiority of primary patency after DCB angioplasty over POBA sustained throughout 5 years (Kaplan–Meier point estimate at 5 years: 61.4% vs. 53.5%; log-rank *p* = 0.040). The advantage of DCB over POBA decreased over time (Fig. 2A, Supplementary Table 2). Post hoc analysis could not demonstrate any significant interaction of subgroups with the treatment effect (overall loss of primary patency: HR 0.55 (95%CI 0.32 to 0.94) in favor of DCB; *p* = 0.03) (Fig. 3). The declining gap between DCB and POBA is especially obvious in the Kaplan–Meier estimates for freedom from CD-TLR after 2 years up to 5 years (Fig. 2B). Freedom from CD-TLR at 5 years did not differ significantly between groups (DCB 82.1% vs. POBA 73.7%, log-rank *p* = 0.050) (Fig. 2B). Freedom from TVR was 68.1% (95%CI 54.4 to 78.5) with DCB and 69.5% (95%CI 56.6 to 79.3) with POBA (log-rank *p* = 0.37).

**Fig. 4:**
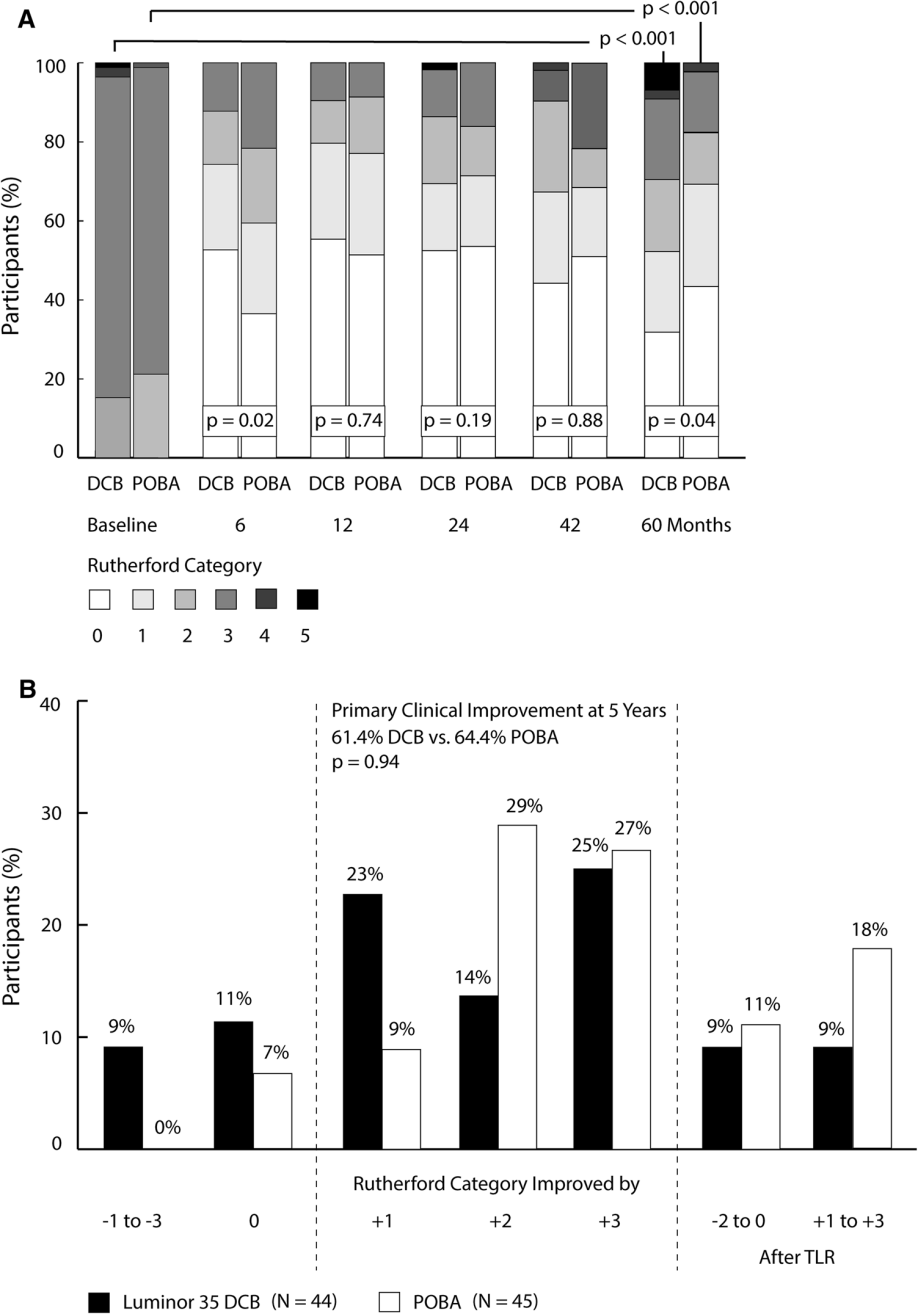
Clinical improvement according to Rutherford classification. **A** Change in Rutherford category from baseline to follow-ups (participants who underwent TLR included), and **B** clinical improvement at 5 years. The boxed *p*-values concern differences in change from baseline between the DCB and the POBA group. Primary clinical improvement applied if the Rutherford category declined by at least one level without preceding target lesion revascularization. *DCB* drug-coated balloon, *POBA* plain old balloon angioplasty, *TLR* target lesion revascularization

Rutherford classification of participants remained significantly shifted to more favorable categories compared to baseline in both groups. However, with inclusion of participants who had undergone CD-TLR in the analysis, the POBA group showed a more advantageous distribution of categories than the DCB group (Fig. 4A). If CD-TLR participants were excluded from the comparison, primary clinical improvement did not differ between groups (DCB 61.4% vs. POBA 64.4%, *p* = 0.94) (Fig. 4B). Secondary clinical improvement was 70.5% (31 of 44 participants) and 82.2% (37 of 45 participants) after DCB angioplasty and POBA, respectively (*p* = 0.22). Primary and secondary hemodynamic improvement was similar between groups (DCB 45% [12 of 31] vs. POBA 39% [15 of 33], *p* = 0.62, and DCB 48% [15 of 31] vs. POBA 70% [23 of 33 participants], *p* = 0.13, respectively).

### Safety Outcomes through 5 Years

**Fig. 5:**
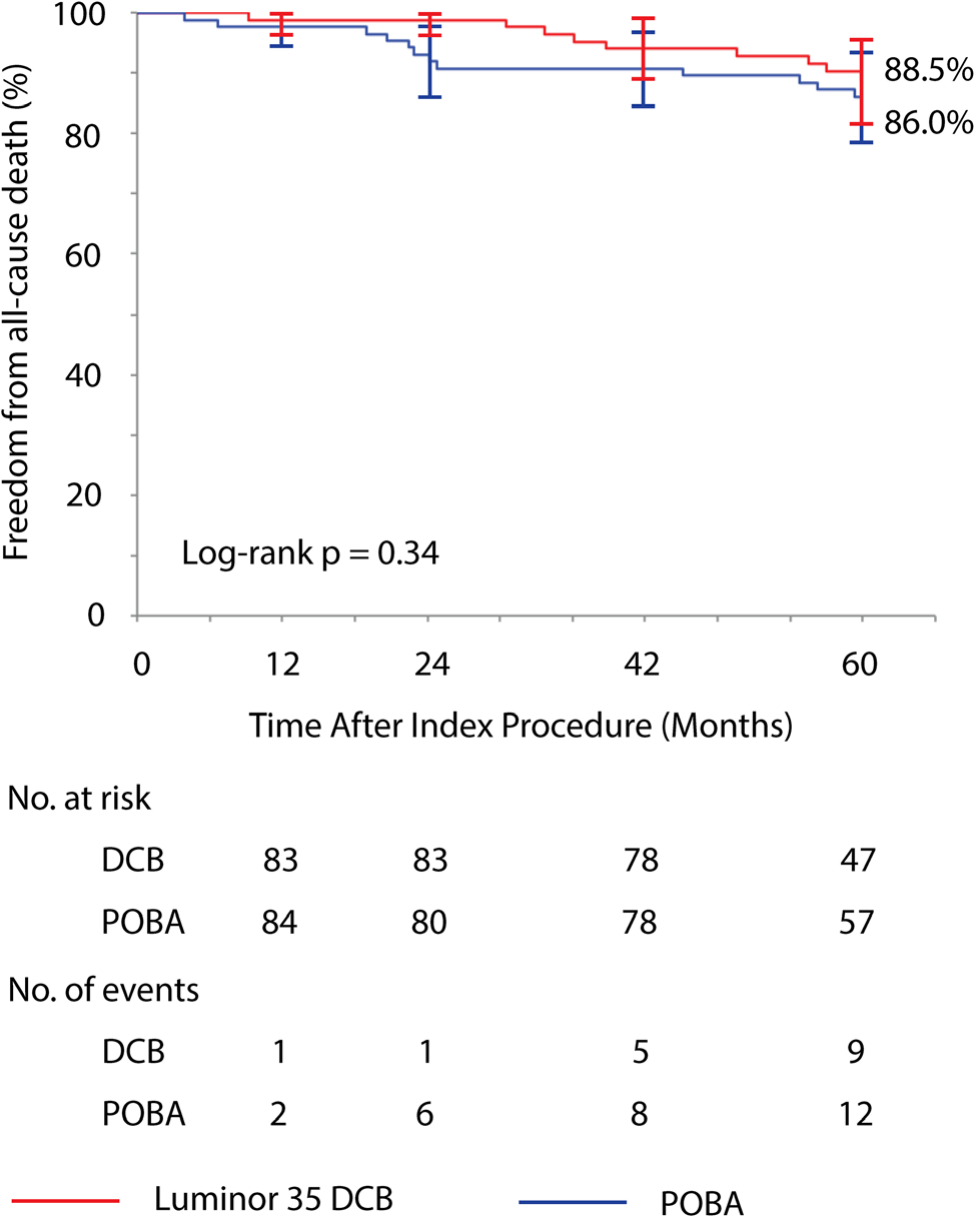
Survival through 5 years. Kaplan–Meier curves show freedom from all-cause death after Luminor 35 DCB angioplasty or standard balloon angioplasty through 5 years. At 60 months, standard error was 3.6% and 3.8% in the DCB and POBA group, respectively. Bars represent 95% confidence intervals. *DCB* drug-coated balloon, *POBA* plain old balloon angioplasty

Incidence of restenosis, target limb amputation, or all-cause death at 5 years did not differ significantly between groups. Eleven percent (9 of 80) of the participants died after DCB angioplasty, and 16% (14 of 86) after POBA. No death occurred within 30 days after the procedure and no significant difference was seen regarding causes of deaths (Table 2). Kaplan–Meier estimate of freedom from all-cause death at 5 years was similar between groups (DCB 88.5% vs. POBA 86.0%, log-rank p-value 0.34) (Fig. 5, Supplementary Table 2).

**Table 2:** Safety outcomes at 5 years^a^

	Luminor 35 DCB (*N* = 85)	POBA (*N* = 86)	*P* Value
All-cause death	9/80 (11)	14/86 (16)	0.38
Within 30 days	0/9	0/14	
Undetermined cause^b^	2/9 (22)	3/14 (21)	> 0.99
Cardiovascular-related^b^	2/7 (29)	5/11 (45)	0.64
Cardiac-related	1/7 (14)	4/11 (36)	0.60
Stroke	1/7(14)	1/11 (9)	> 0.99
Non-cardiovascular-related^b^	5/7 (71)	6/11 (55)	0.64
Neoplasms	2/7 (29)	1/11 (9)	0.53
Respiratory failure	0/7	2/11 (18)	0.50
Multiorgan failure	1/7 (14)	1/11 (9)	> 0.99
Infection/sepsis	1/7 (14)	1/11(9)	> 0.99
Diabetic coma	1/7 (14)	0/11	0.39
Suicide	0/7	1/11 (9)	> 0.99
Major target limb amputation	0/49	1/51 (2)	> 0.99
Minor target limb amputation	0/49	1/51 (2)	> 0.99
Binary restenosis	23/45 (51)	35/54 (65)	0.22
CD-TLR	10/49 (20)	18/51 (35)	0.12
Any TVR^c^	19/53 (36)	21/51 (41)	0.69

Data are counts (percentage)

^a^Sixty-five months were set as cutoff for analysis

^b^Causes of death were adjudicated by an independent data safety monitoring board. None of the deaths were assessed as device- or procedure-related

^c^TVR included TLR and non-TLR TVR

*DCB* drug-coated balloon, *POBA* plain old balloon angioplasty, *CD-TLR*, clinically driven target lesion revascularization, *TVR* target vessel revascularization

## Discussion

The present study demonstrates Luminor® 35 DCB angioplasty of femoropopliteal lesions to be superior in terms of primary patency compared to POBA throughout 5 years, however, with a declining gap. Regarding freedom from CD-TLR, the advantage of DCB was still numerically apparent but no longer significant. Over the long term, treatment groups were largely similar with regards to clinical and hemodynamic improvement. Even at 5 years, all-cause mortality did not differ between both treatment groups.

Although, advantage of DCB over POBA decreased over time, benefit of DCB concerning prevention of restenosis is still evident over the long term. Our finding supports previous reports on the final clinical outcome of CD-TLR. Both the IN-PACT SFA study [7] and the AcoArt study [8] reported on favorable results on freedom from CD-TLR with DCB compared to POBA after 5 years (74.5% vs 65.3%, *p* = 0.02 and 77.5% vs. 59.1%, *p* < 0.001, respectively). Five-year cumulative incidence of freedom from CD-TLR after DCB in the large real world IN.PACT Global study [13] was somewhat lower (69.4%).

In the EffPac trial, the difference in CD-TLR between DCB and POBA at 5 years could no longer be considered significant as it has been at 42 months [14]. Notably, incidences of freedom from CD-TLR in both EffPac groups, DCB as well as POBA, were considerably higher as in the IN-PACT SFA and the AcoArt studies. Differences in effectiveness may be based on lesion complexity and treatment strategy [3]. In the AcoArt study, the share of participants with critical limb-threatening ischemia (CLTI) and diabetes was considerably higher compared to EffPac. In addition, lesions were longer and about one-quarter were in-stent restenosis. In the IN.PACT global registry, about 80% of participants were assigned to a “broader use” group with longer lesions and a high share of in-stent restenosis (21%). In the IN-PACT SFA randomized study, participants and lesions were closer to the EffPac trial, however, bailout stenting occurred less frequently. In both the AcoArt and the IN.PACT SFA study, pre-dilation was less frequently conducted in POBA than in DCB participants, which might have contributed to poorer outcomes after POBA. Vessel preparation is crucial for long-term outcomes. Therefore, the EffPac protocol required pre-dilation with POBA mandatorily in both study arms and the randomization was performed after pre-dilatation to assure that both study arms were pre-dilated equally.

Regardless of lesion characteristics and treatment strategies, effectiveness of DCB angioplasty might also be determined by the coating design (excipient, drug, drug dose, coating technology). In vitro tests showed that, depending on the adherence of the coating, material gets partly lost already during removal of the protective cover of the balloon [15] and subsequently, during transfer to the target lesion [16]. Paclitaxel loss during removal of the protective cover was reported to be considerably higher with IN.PACT Admiral than with Luminor® 35 DCB angioplasty [15]. However, conversely, in vitro abrasion of the coating from the uninflated balloon was found to be significantly more severe with Luminor® 35 [16]. Although a strong in vitro adherence of the coating is not inevitably associated with increased in vivo drug transfer into the vessel wall, drug loss might result in both unreliable clinical efficacy and increased risk of distal particulate embolization of amorphous- or crystalline-like material. Downstream loss of paclitaxel is held accountable for fibroid necrosis and inflammation in small arteries [17] that might be associated with slow flow [18]. However, in the EffPac trial, no treatment interaction of previously identified risk factors of slow flow, such as chronic total occlusion or poor distal runoff [19] became clinically evident. However, this might be due to the limited power of our subgroup analysis. In addition, particular embolization might be more critical in patients with long lesions and CLTI.

Against the background of the controversial debate on long-term safety of paclitaxel in peripheral interventions [4, 20–22] we completed the vital status in almost all participants. In accordance with a recent meta-analysis on paclitaxel-coated devices for predominately claudicant participants that included 9 randomized controlled trials with 5-year results [23], all-cause mortality in the EffPac trial did not differ between treatment groups. At 5 years, the meta-analysis found a somewhat higher all-cause mortality of 18.8% after paclitaxel-coated devices and a similar all-cause mortality of 15.9% after POBA (*p* = 0.08). There was no significant heterogeneity between studies.

Similar to the survival curves for freedom from TLR that have been reported previously [7, 8], in the EffPac study the advantage of DCB angioplasty over POBA decreased over time. This was mainly driven by a frequent loss of patency after POBA within the first 12 months. In contrast, DCB angioplasty considerably slowed down the decline of patency loss. We do not suspect any late catch-up phenomenon from continuous neointimal growth known from drug-eluting stents in the coronary arteries [24] because the half-live of paclitaxel is reported with 45 days after a single local administration with DCB [25]. According the “leaving nothing behind” principle the avoidance of primary stenting may eliminate another trigger for neointimal hyperplasia.

As a matter of fact, we are seeing a declining gap between DCB and POBA throughout 5 years. The Kaplan–Meier curves for primary patency and freedom from CD-TLR are clearly demonstrating the vanishing effectiveness of DCB compared to POBA over time  (Fig. 2). Although there is still a significant difference in primary patency between DCB angioplasty and POBA at 5 years, it can be assumed that this effect will disappear after some time.

Finally, post-hoc subgroup analysis generates the hypothesis that loss of patency in patients at older age is similar with DCB and POBA (Fig. 3). This might be due to a reduced neointimal proliferation with age. Accordingly, a previous EffPac multivariable analysis showed less LLL at 6 months with advanced age independent of whether DCB or POBA was used [26]. Whether patients with poor distal runoff benefit more from DCB remains to be assessed.

### Limitations

This trial has limitations. First, although vital status was obtained almost entirely, clinical and ultrasonography follow-up was completed in only about half of the participants. Second, the study was not powered to assess a difference in long-term all-cause mortality between the treatment groups. Finally, in this study we evaluated the Luminor® 35 DCB. Thus, results cannot be transferred automatically to other DCB types.

### Conclusion

Long-term follow-up of the EffPac trial showed superiority in terms of primary patency after femoropopliteal Luminor® 35 DCB angioplasty compared to POBA over a period of 5 years. This finding was reflected by freedom from TLR, however, no longer with a significant difference. No safety signal occurred. From this, we can conclude that femoropopliteal Luminor® 35 DCB angioplasty is a sustainably efficacious and safe treatment approach.

## Supplementary Information

Below is the link to the electronic supplementary material.

Supplementary file1 (DOC 74 KB)
