# Supplementary material for: Long-Term Effectiveness and Safety of Femoropopliteal Drug-Coated Balloon Angioplasty : 5-Year Results of the Randomized Controlled EffPac Trial
Source: Cardiovasc Intervent Radiol. 2022 Sep 11;45(12):1774–83. doi: 10.1007/s00270-022-03265-1 (PMC9705448; doi:10.1007/s00270-022-03265-1)
Supplement: Supplementary file 1 — Supplementary file1 (DOC 74 KB) [file 270_2022_3265_MOESM1_ESM.doc]

| **Supplementary Table 1** EffPac trial eligibility criteria |
| --- |
| **Inclusion criteria**   1. Age ≥ 18 years 2. Subject must agree to undergo the 6-month angiographic and clinical follow-up (at 12- and 24 months post-procedure) 3. Peripheral vascular disease Rutherford class 2-4 4. De novo stenotic/ re-stenotic lesion or occlusive lesions in the superficial femoral (SFA) and/or popliteal arteries (PA) 5. If the index lesion is re-stenotic, the prior PTA must have been >30 days prior to treatment in the current study 6. ≥70% diameter stenosis or occlusion 7. Target lesion length: ≤15 cm 8. Only one lesion per limb and per patient can be treated 9. ≥ one patent infrapopliteal run-off artery to the index limb foot 10. Successful endoluminal guidewire passage through the target lesion 11. Pre-dilatation prior to randomization 12. Life expectancy, in the investigators’ opinion of at least one year 13. Subject can verbally acknowledge and understand the aim of this trial and is willing and able to provide informed consent. |
| **Exclusion criteria**   1. Previous surgery in the target vessel 2. Patients who require a PTA balloon catheter in diameter size 4 mm or in diameter size greater 7 mm. 3. Major amputation in the same limb as the target lesion 4. Acute myocardial infarction within 30 days before intervention 5. Severely calcified target lesions in the SFA/PA resistant to PTA 6. Subjects requiring different treatment or raising serious safety concern regarding the procedure or the required medication 7. Women of childbearing potential except women with the following criteria:    1. post-menopausal (12 months natural amenorrhea or 6 months amenorrhea with serum FSH > 40mlU/ml)    2. sterilization after bilateral ovariectomy with or without hysterectomy    3. using an effective method of birth control for the duration of the trial: implants, injectables, combined oral contraceptives, intrauterine device (in place for a period of at least 2 months prior to screening) and with negative serum pregnancy test    4. sexual abstinence    5. vasectomy partner 8. Pregnant and nursing women 9. Acute thrombus aneurysm in the index limb or vessel 10. In-stent restenosis in the target lesion 11. Renal insufficiency with a serum creatinine >2.0 mg/dL at baseline 12. Platelet count <50 G/l or >600 G/l at baseline 13. Known hypersensitivity or contraindication to contrast agent that cannot be adequately pre-medicated 14. Subjects with known allergies against paclitaxel 15. Subjects with intolerance to antiplatelet, anticoagulant, or thrombolytic medications that would be administered during the trial 16. Dialysis or long-term immunosuppressant therapy 17. Current participation (or within the last 3 months) in another interventional study. |

| **Supplementary Table 2** Kaplan-Meier point estimates of primary patency, freedom from clinically driven TLR, and freedom from all-cause deathl through 5 years | | |
| --- | --- | --- |
|  | **DCB** | **POBA** |
| Primary patency |  |  |
| 6 months | 100% | 92.1% (95%CI 86.0 to 98.2) |
| 1 year | 94.7% (95%CI 89.6 to 99.8) | 71.7% (95%CI 61.4 to 82.0) |
| 2 years | 90.3% (95%CI 83.4 to 97.1) | 63.6% (95%CI 52.3 to 74.9) |
| 42 months | 68.9% (95%CI 56.6 to 81.2) | 60.0% (95%CI 48.3 to 71.7) |
| 5 years | 61.4% (95%CI 47.9 to 75.0) | 53.5% (95%CI 40.9 to 66.1) |
| Freedom from CD-TLR |  |  |
| 6 months | 100% | 94.8% (95%CI 89.8 to 99.8) |
| 1 year | 98.7% (95%CI 96.2 to 100) | 81.6% (95%CI 72.9 to 90.3) |
| 2 years | 97.2% (95%CI 93.3 to 100) | 78.1% (95%CI 68.5 to 87.7) |
| 42 months | 88.3% (95%CI 80.1 to 96.5) | 78.1% (95%CI 68.5 to 87.7) |
| 5 years | 82.1% (95%CI 71.9 to 92.3) | 73.7% (95%CI 62.8 to 84.5) |
| Survival |  |  |
| 6 months | 100% | 98.8% (95%CI 96.6 to 100) |
| 1 year | 98.8% (95%CI 96.5 to 100) | 97.7% (95%CI 94.5 to 100) |
| 2 years | 98.8% (95%CI 96.5 to 100) | 93.0% (95%CI 87.6 to 98.4) |
| 42 months | 94.0% (95%CI 88.9 to 99.1) | 90.7% (95%CI 84.6 to 96.8) |
| 5 years | 88.5% (95%CI 81.4 to 95.7) | 86.0% (95%CI 78.5 to 93.3) |

*CD-TLR* clinically driven target lesion revascularization*, DCB* drug-coated balloon, *POBA* plain old balloon angioplasty

**References**

1. Teichgräber U, Aschenbach R, Scheinert D et al. The effectiveness of the paclitaxel-coated Luminor® balloon catheter versus an uncoated balloon catheter in superficial femoral and popliteal arteries in preventing vessel restenosis or reocclusion: study protocol for a randomized controlled trial. Trials. 2016;17(1):528.

2. Teichgraber U, Lehmann T, Aschenbach R et al. Drug-coated Balloon Angioplasty of Femoropopliteal Lesions Maintained Superior Efficacy over Conventional Balloon: 2-year Results of the Randomized EffPac Trial. Radiology. 2020;16:191619.
